# Seasonal Dynamics of Microbial Communities in PM_2.5_ and PM_10_ from a Pig Barn

**DOI:** 10.3390/ani15081116

**Published:** 2025-04-12

**Authors:** Qian Tang, Minyang Zhang, Lili Yu, Kaidong Deng, Huihua Mao, Jingwen Hu, Chuang Wang

**Affiliations:** 1 College of Animal Science and Food Engineering, Jinling Institute of Technology, Nanjing 210038, China; tangqian@jit.edu.cn (Q.T.); yll@jit.edu.cn (L.Y.); kdeng@jit.edu.cn (K.D.); mhh@jit.edu.cn (H.M.); 15189011628@163.com (J.H.); w22715815@163.com (C.W.); 2 Research Center for Livestock Environmental Control and Smart Production, College of Animal Science and Technology, Nanjing Agricultural University, Nanjing 210095, China

**Keywords:** pig house, particulate matter, microbial composition, season

## Abstract

Modern, intensive, high-density farming practices cause elevated concentrations of particulate matter (PM) inside livestock barns. PM in livestock barns is predominantly biological, hence, it contains abundant microorganisms. Understanding the microbial composition of PM is crucial for assessing the hazards of air emitted from livestock barns. PM_10_ and PM_2.5_ from a pig barn were collected in winter and spring, and morphological, chemical, and microbial analyses were performed. The PM samples exhibit diverse morphological characteristics. The top three elements detected in the PM samples were O, C, and Si. Other elements, including N, Al, K, Mg, Ca, Na, Zn, P, W, Ba, Fe, S, Cl, and Ti, were also identified in these samples. For bacterial α diversity, the Sobs and Chao1 indices for PM_10_ were significantly higher than those for PM_2.5_ in winter (*p* < 0.05), and in spring, the ACE index for PM_10_ was significantly higher than that for PM_2.5_ (*p* < 0.05). For fungal α diversity, the Shannon index for PM_10_ was significantly higher than that for PM_2.5_ in winter (*p* < 0.01), and in spring, the Ace index for PM_10_ was significantly higher than that for PM_2.5_ (*p* < 0.05). The β diversity results indicate that season, rather than the particle size, had a significant effect on the microbial composition in the PM samples. A total of seven bacterial pathogen genera and 16 fungal allergen genera were identified in PM samples. In winter, the relative abundances of total bacterial pathogens and fungal allergens in PM_2.5_ were higher than those in PM_10_. In contrast, the relative abundance of fungal allergens in PM_10_ was higher in spring than in winter. This study provides a comprehensive characterization of PM from a pig barn across the particle sizes and seasons.

## Simple Summary

Characterizing the microbial composition of particulate matter (PM) in pig barns is essential for assessing potential health risks to both livestock and farmers. The bacterial and fungal composition of different-sized PM samples (PM_2.5_ and PM_10_) were identified across spring and winter. The results showed that the season, rather than the particle size, had a significant impact on the microbial community composition of the PM samples. In total, seven pathogenic bacterial genera and 16 fungal allergen genera were identified across different seasons. In winter, the relative abundances of total bacterial pathogens and fungal allergens contained in PM_2.5_ were both higher than those in PM_10_. These findings indicate that, in winter, PM_2.5_ may pose a greater threat to animals and humans than PM_10_. This study improves our understanding of the characteristics of PM from livestock barns across the particle sizes and seasons.

## 1. Introduction

Livestock barn-derived particulate matter (PM) impairs animal growth performance and poses health risks to farm workers [1,2]. Nonfarming residents who are constantly exposed to livestock farm-emitted PM are also at increased risk for respiratory diseases and atopic sensitization [3]. PM from livestock barns is derived from feed, feces, urine, dander, bedding, skin, hair, and other sources. PM from livestock barns contains more abundant microorganisms. In particular, pathogenic microorganisms in PM pose a threat to both animals and farmers. These microorganisms can also be discharged through the ventilation system, endangering the surrounding environment, and impact the respiratory health of people living in areas with high livestock density [3].

The main physiological function of the respiratory system is gas exchange between the body and the air, which means that the respiratory tract is exposed to the outside environment. In the past, it was believed that the respiratory tract was sterile within a healthy individual. However, microbial sequencing has confirmed the presence of microorganisms in both the upper and lower respiratory tract [4,5]. Various microorganisms can even be found in the upper respiratory tract of newborns [6].

The deposition site of PM in the respiratory tract is primarily determined by its aerodynamic diameter. PM_10_ refers to PM that is less than 10 μm in size (i.e., coarse particles), and PM_2.5_ refers to PM that is less than 2.5 μm in size (i.e., fine particles). For humans, particles larger than 5 μm are blocked in the upper respiratory tract, nose, pharynx, and throat, but PM_2.5_ can enter the lower respiratory tract, trachea, bronchi, and even alveoli while breathing [7]. Previous analyses of bacterial aerosol similarities across different PM size fractions and the piglet respiratory tract revealed that airborne bacterial aerosols of 1.1–3.3 µm were highly similar to bacteria in the lower respiratory tract (bronchus and lung) of piglets, while aerosols >3.3 µm were similar to those in the upper respiratory tract (mouth and nose) [8]. This result indicates that the microbial composition of PM was similar to that of the respiratory tract.

Respiratory tract-colonizing microbiota maintain a steady state and dynamic balance. If this balance is disrupted, an invasion of pathogenic microorganisms into the respiratory tract may occur, which damages respiratory tract health [9,10]. A recent study reported that *Haemophilus influenzae*, *Klebsiella pneumoniae*, and *Streptococcus pneumoniae* more frequently colonize the upper respiratory tract during lower respiratory tract infections [11]. A recent case–control study in adults revealed that *H. influenzae* and *S. pneumoniae*, together with a low abundance of commensal bacteria, including *Corynebacterium* and *Staphylococcus*, were linked to pneumonia [12]. This homeostasis may be disturbed by environmental pollutants, especially the microorganisms contained in airborne particles. Previous research utilizing mice as an animal model demonstrated that PM_2.5_ from a nursery pig barn altered the pulmonary microbial composition by increasing the relative abundance of Firmicutes and the Firmicutes/Bacteroidetes ratio while reducing the relative abundance of Bacteroidetes [13]. Similarly, Shen et al. reported that PM_2.5_ from broiler barns induced lung inflammation in the birds and changed the lung microbiome, which included 66 different microbial genera [14]. Characterizing airborne microbial composition is therefore essential for understanding respiratory health.

Microbial composition analysis of pig barn PM_2.5_ across seasons demonstrated that temporal variation predominated over other factors in shaping aerosol microbiota [15]. However, the microorganisms in PM of different sizes and during different seasons have not been studied in detail in pig barns. In this study, PM_10_ and PM_2.5_ samples were collected from a lactating sow barn during winter and spring. Morphological and chemical analyses of the PM_2.5_ and PM_10_ samples were performed, and a high-throughput sequencing technique was performed to determine the bacterial and fungal compositions across PM sizes and seasons. Potential bacterial pathogens and fungal allergens were then identified. This study provides a comprehensive understanding of the distribution characteristics of PM from a pig barn across sizes and seasons.

## 2. Materials and Methods

### 2.1. Description of the Study Site

This experiment was conducted at a scaled pig farm in Yancheng City, Jiangsu Province, China (32°85′~34°20′ N, 119°57′~120°45′ E). All experimental procedures were conducted following the guidelines of the Administration of Animal Care and Use and were approved by the Animal Ethics Committee of Jinling Institute of Technology, Nanjing, China. A lactating sow barn was chosen. The total area of this barn was 213 m^2^, with a length of 31.3 m, a width of 6.8 m, a height of 2.4 m, and a central aisle width of 1.0 m. The lactating sow barn was oriented in an east–west direction and was equipped with two rows that each contained 16 farrowing crates. A farrowing crate measures 2.3 m in length, 1.8 m in width, and 1.2 m in height. There were 29 lactating sows and approximately 320 nursing piglets in the pig barn. The front entrance of the pig barn was equipped with wet curtains on both sides, and the back entrance was fitted with fans. Additionally, there were ten windows on the south wall and another ten windows on the north wall. The floor plan of the pig barn is shown in Supplementary Figure S1. The pig barn floor is slatted and contains gaps, and the staff performs manure cleaning once per month. The sows were fed automatically at 10:00 and 14:00. The barn was equipped with six LED lights to provide light from 6:00 to 17:00. Heat lamps were on all day to keep the suckling piglets warm. Mechanical ventilation was the primary method of ventilation inside the house.

### 2.2. Sample Collection

An ambient air particulate matter sampler (Model: Laoying 2030, Qingdao Lonying Environmental Technology Co., Ltd., Qingdao, China), with a sampling flow rate of 100 L/min, was placed in the middle of the pig barn. Before sampling, the glass fiber filter membrane used for collecting particulate matter was pretreated. The membrane was baked in a muffle oven at 450 °C for four hours to remove organic residues. Airborne PM_2.5_ and PM_10_ samples were collected during winter (from 1 February to 20 February 2021) and spring (from 1 May to 20 May 2021). The sampling duration for each fiber membrane was 23 h, from 7:00 a.m. to 6:00 a.m. the following day. A total of twenty filter membranes were collected per season and subsequently preserved at −80 °C for further analysis.

### 2.3. Characteristics of PM_2.5_ and PM_10_

Morphological observation and chemical analysis of the PM_2.5_ and PM_10_ samples were performed as described previously [16]. The chemical analyses by X ray spectroscopy were performed on individual particles from each filter, with four randomly selected particles analyzed per filter. Each type of filter had three replicates. Morphological features were observed via a scanning electron microscope (SEM), (Hitachi, Tokyo, Japan). Chemical elements were identified via an energy-dispersive X-ray spectrometer (EDS, Bruker, Germany) attached to the SEM. SEM-EDS analysis was performed manually under the following conditions for visualizing the PM samples: an accelerating voltage of 20 keV, a working distance of 10 mm, a magnification of 4000×, and an X-ray acquisition time of 60 s per particle.

### 2.4. DNA Extraction and PCR Amplification

Five PM_10_ and five PM_2.5_ samples were randomly selected from both spring and winter seasons for bacterial and fungal sequencing analysis. DNA extraction from the PM samples and subsequent PCR amplification were performed as described previously [15]. Half of each filter sample was cut into small pieces. DNA from the PM_2.5_ and PM_10_ samples was extracted via a Power-Soil DNA isolation kit (MoBio Laboratories, Carlsbad, CA, USA) according to the manufacturer’s instructions. For bacterial composition analysis, the primers 338 F (5′-ACTCCTACGGGAGGCAGCAG-3′) and 806 R (5′-GGACTACHVGGGTWTCTAAT-3′) [17] were used to amplify the V3-V4 hypervariable region of the 16S rRNA gene. For fungal composition analysis, the primers ITS1F (5′-CTTGGTCATTTAGAGGAAGTAA-3′) and ITS2 (50-TGCGTTCTTCATCGATGC-3′) [18] were used to amplify the ITS1 region of the fungal ITS rRNA gene. Purified PCR products were paired-end sequenced on an Illumina MiSeq PE300 platform at Majorbio Bio-Pharm Technology Co. Ltd. (Shanghai, China).

### 2.5. Sequence Analyses

Raw sequences were removed if they were shorter than 200 bp, had a poor quality score (≤20), contained ambiguous bases, or did not exactly match primer sequences and barcode tags. Qualified reads were separated via the sample-specific barcode sequences and trimmed using Illumina Analysis Pipeline Version 2.6. Operational taxonomic units (OTUs) were counted for all samples at a similarity level of 97% using the UCLUST function in QIIME to generate rarefaction curves and calculate the richness and diversity indices. The taxonomy of each gene sequence was analyzed using the RDP Classifier algorithm against the Silva database. The α-diversity indices (observed species, ACE, Simpson, Shannon, and Chao 1 indices) were calculated for each sample, and β diversity was analyzed via nonmetric multidimensional scaling (NMDS) and principal coordinate analysis (PCoA) at the OTU level on the basis of the unweighted UniFrac distance matrix, which was constructed to determine differences in microbial community structure between groups.

### 2.6. Statistical Analysis

Statistical analyses were performed with GraphPad Prism version 9.01 (GraphPad Software, Inc., La Jolla, CA, USA). Significant differences between the two groups were analyzed with a Student’s *t* test if the data were normally distributed or Mann–Whitney U test if the data were not normally distributed. Data from more than two groups were analyzed by one-way ANOVA followed by Tukey’s test or a Kruskal–Wallis test according to if the data were normally distributed. All *p*-values less than 0.05 were regarded as statistically significant.

## 3. Results

### 3.1. Morphological and Chemical Analysis of PM_2.5_ and PM_10_

**Figure 1:**
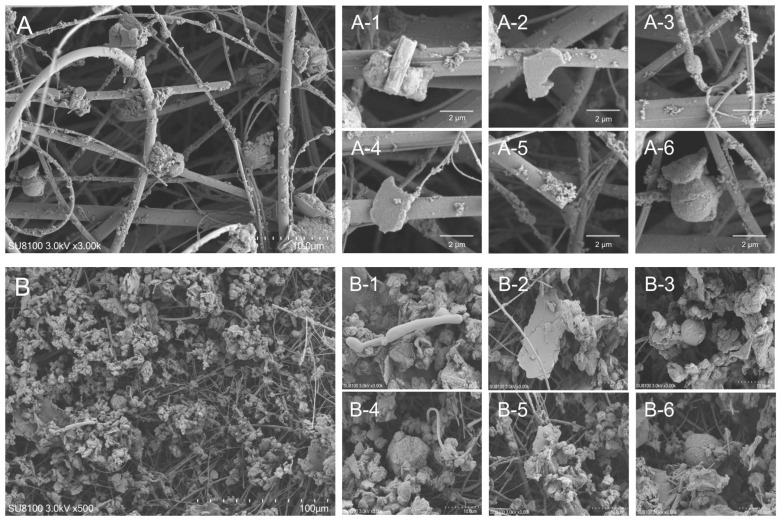
Typical ultrafine structure of PM_2.5_ and PM_10_ samples from a lactating sow barn. (**A**) PM_2.5_ samples, scale bar = 10 μm; (**A-1**–**A-6**) SEM image from X-ray analysis of a single particle of PM_2.5_, scale bar = 2 μm. (**B**) PM_10_ samples, scale bar = 100 μm; (**B-1**–**B-6**) SEM image from X-ray analysis of a single particle of PM_10_, scale bar = 10 μm.

**Figure 2:**
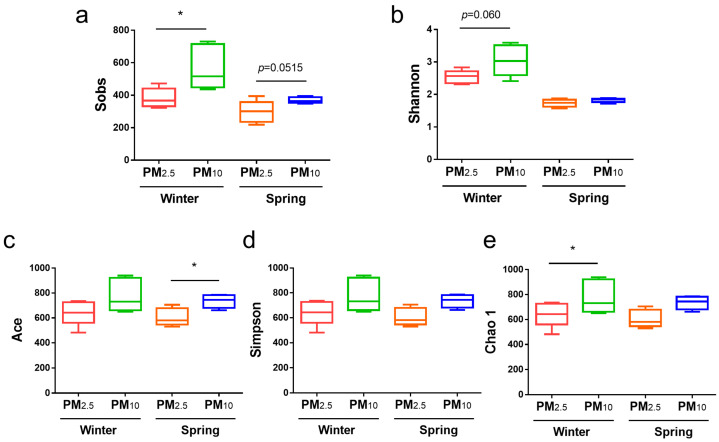
Bacterial α diversity of PM_2.5_ and PM_10_ in winter and spring, respectively. (**a**) Sobs; (**b**) Shannon; (**c**) Ace; (**d**) Simpson; and (**e**) Chao 1 indices. * *p* < 0.05.

**Figure 3:**
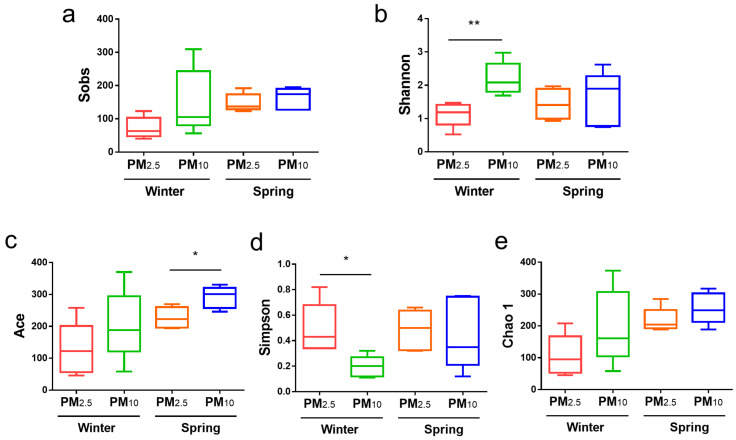
Fungal α diversity of PM_2.5_ and PM_10_ in winter and spring, respectively. (**a**) Sobs; (**b**) Shannon; (**c**) Ace; (**d**) Simpson; and (**e**) Chao 1 indices. * *p* < 0.05, ** *p* < 0.01.

**Figure 4:**
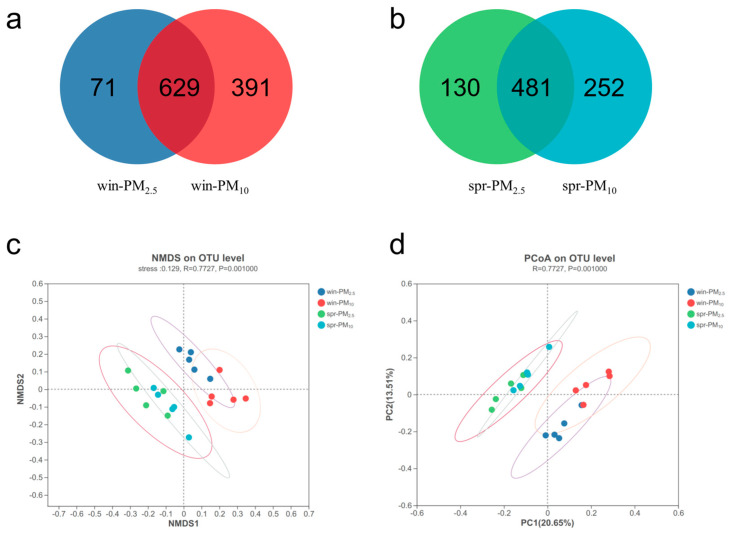
Bacterial Venn diagram and β diversity of PM_2.5_ and PM_10_ in winter and spring, respectively. (**a**) A Venn diagram of PM_2.5_ and PM_10_ in winter; (**b**) a Venn diagram of PM_2.5_ and PM_10_ in spring; (**c**) nonmetric multidimensional scaling (NMDS) of the OTU levels of PM_2.5_ and PM_10_ in winter and spring, stress = 0.129; (**d**) principal coordinate analysis (PCoA) of the OTU levels of PM_2.5_ and PM_10_ in winter and spring, respectively.

**Figure 5:**
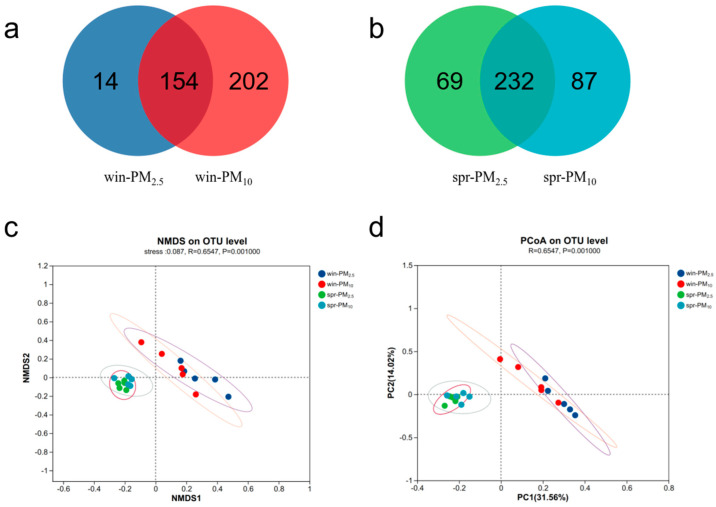
Fungal Venn diagram and β diversity of PM_2.5_ and PM_10_ in winter and spring, respectively. (**a**) A Venn diagram of PM_2.5_ and PM_10_ in winter; (**b**) a Venn diagram of PM_2.5_ and PM_10_ in spring; (**c**) nonmetric multidimensional scaling (NMDS) of the OTU levels of PM_2.5_ and PM_10_ in winter and spring, stress = 0.087; (**d**) principal coordinate analysis (PCoA) of the OTU levels of PM_2.5_ and PM_10_ in winter and spring, respectively.

**Figure 6:**
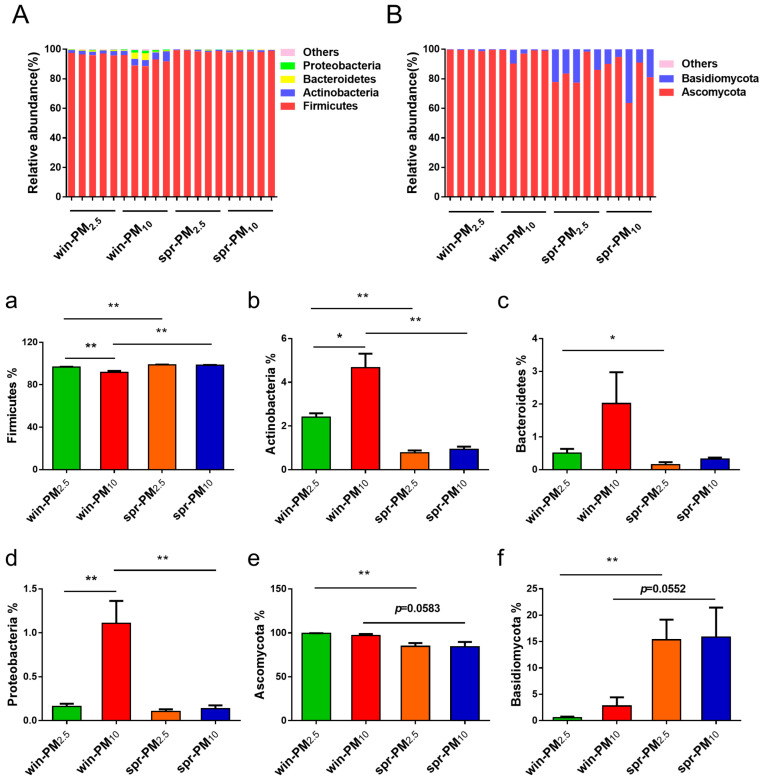
Phylogenetic classification of bacterial communities (**A**) and fungal communities (**B**) at the phylum level. Bacterial phyla with a relative abundance higher than 0.10% and those showing significant differences are presented (**a**–**d**). Fungal phyla with a relative abundance higher than 0.10% and those with significant differences are shown (**e**,**f**). * *p* < 0.05, ** *p* < 0.01.

The typical morphologies of the particles are shown in Figure 1. In the PM_2.5_ samples, strip-shaped particles (Figure 1(A-1)), rod-shaped particles (Figure 1(A-3)), angular, fragmented, and flaky particles (Figure 1(A-2,A-4)), highly aggregated rice-like particles (Figure 1(A-5)), and spherical particles (Figure 1(A-6)) were observed. In the PM_10_ samples, elongated particles (Figure 1(B-1)), wrinkled flaky particles (Figure 1(B-2,B-5)), spherical particles of varying diameters (Figure 1(B-3,B-6)), and gravel-like particles (Figure 1(B-4)) were identified. The mass percentages of chemical composition of PM_2.5_ and PM_10_ samples in winter and spring (Supplementary Figure S2) were quantified. The top three elements detected in the PM samples were O, C, and Si, respectively. Other elements, including N, Al, K, Mg, Ca, Na, Zn, P, W, Ba, Fe, S, Cl, and Ti, were also identified from these samples (Table 1).

### 3.2. Taxonomic Diversity and Composition of Bacteria and Fungi in PM_2.5_ and PM_10_

Bacterial α diversity in the PM_10_ and PM_2.5_ samples from winter and spring was compared using the Shannon and Simpson indices (diversity) and the Sobs, chao 1, Ace indices (richness) (Figure 2). In terms of bacteria, in winter, the Sobs and Chao1 indices for PM_10_ were significantly higher than those for PM_2.5_ (*p* < 0.05) (Figure 2a,e); in spring, the Ace index for PM_10_ was significantly higher than that for PM_2.5_ (*p* < 0.05) (Figure 2c). In addition, the Sobs index for PM_10_ in spring was slightly higher than that for PM_2.5_ (*p* = 0.0515) (Figure 2a), and the Shannon index for PM_10_ in winter was higher than that for PM_2.5_, but no significant differences were detected (Figure 2b).

Fungal α diversity in the PM_10_ and PM_2.5_ in samples from winter and spring was also compared (Figure 3). In terms of fungi, in winter, the Shannon index for PM_10_ was significantly higher than that for PM_2.5_ (*p* < 0.01) (Figure 3b), and the Simpson index was significantly lower than that for PM_2.5_ (*p* < 0.01) (Figure 3d). In spring, the Ace index for PM_10_ was significantly higher than that for PM_2.5_ (*p* < 0.05) (Figure 3c). There were no significant differences among the other groups (*p* > 0.05).

Venn diagram analysis revealed 700 bacterial OTUs in PM_2.5_ and 1020 OTUs in PM_10_ in winter, with a total of 629 shared OTUs (Figure 4a). In spring, there were 611 bacterial OTUs in PM_2.5_ and 733 OTUs in PM_10_, with a total of 481 shared OTUs (Figure 4b).

Venn diagram analysis revealed 168 fungal OTUs in PM_2.5_ and 356 OTUs in PM_10_ in winter, with a total of 154 shared OTUs (Figure 5a). In spring, there were 301 fungal OTUs in PM_2.5_ and 319 OTUs in PM_10_, with a total of 232 shared OTUs (Figure 5b). NMDS and PCoA analyses were performed to assess the β diversity. The bacterial or fungal assemblage clearly differed between spring and winter, whereas no apparent separation was detected between the PM_2.5_ and PM_10_ samples within one season (Figure 4 and Figure 5c,d). The β diversity results indicated that season, rather than particle size, had a significant effect on microbial community composition in the PM samples.

### 3.3. Bacterial Assemblage Composition in PM_2.5_ and PM_10_

A total of 23 different bacterial phyla were identified (Figure 6A). The phylum Firmicutes had the highest relative abundance, accounting for 96.40%, followed by Actinobacteria with 2.19%, Bacteroidetes with 0.75%, and Proteobacteria with 0.38%.

In winter, the relative abundance of the phylum Firmicutes was significantly higher in PM_2.5_ than in PM_10_ (*p* < 0.01) (Figure 6a). Additionally, in winter, the relative abundances of Actinobacteria and Proteobacteria in PM_10_ were significantly higher than those in PM_2.5_ (*p* < 0.05 and *p* < 0.01, respectively) (Figure 6b,d). However, no significant difference in these phyla were observed between PM_2.5_ and PM_10_ in spring (*p* > 0.05).

**Figure 7:**
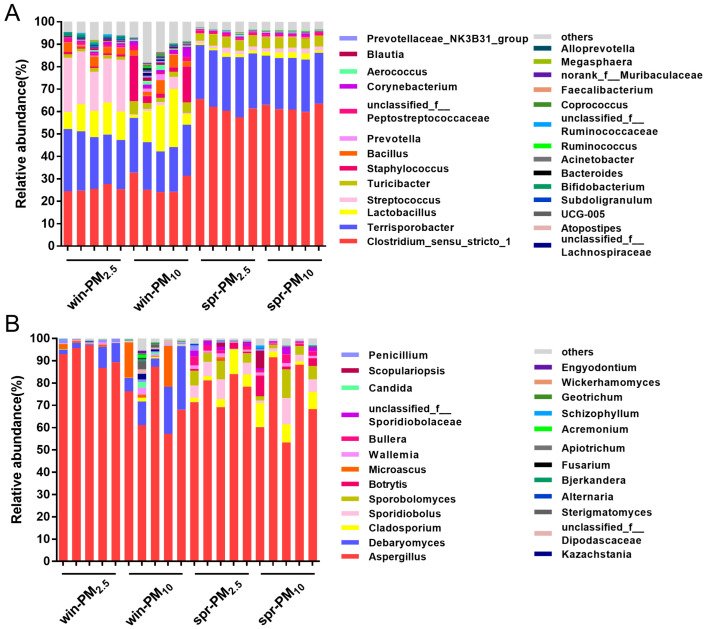
Phylogenetic classification of bacterial communities (**A**) and fungal communities (**B**) at the genera level. The genera with a relative abundance higher than 0.01% are listed.

Next, the differences in bacterial phyla for PM_10_ and PM_2.5_ were analyzed across different seasons. For PM_2.5_, the relative abundance of the phylum Firmicutes was significantly higher in spring than in winter (*p* < 0.01) (Figure 6a). Conversely, the relative abundances of the phylum Actinobacteria and Bacteroidetes were significantly higher in winter than in spring (*p* < 0.05) (Figure 6b,c). For PM_10_, the relative abundance of the phylum Firmicutes was significantly higher in spring than that in winter (*p* < 0.01) (Figure 6a). Furthermore, the relative abundances of the phylum Actinobacteria and Proteobacteria in the PM_10_ samples were significantly higher in winter than in spring (*p* < 0.01) (Figure 6b,d). The bacterial genera with average relative abundances higher than 0.01% are listed in Figure 7A. The different bacterial genera present in PM_2.5_ and PM_10_ during winter and spring seasons are shown in Supplementary Figure S3.

### 3.4. Fungal Assemblage Composition in PM_2.5_ and PM_10_

Seven fungal phyla were identified from the samples (Figure 6B). Together, Ascomycota and Basidiomycota accounted for 99.96% of fungi. Among them, the phylum Ascomycota had the highest relative abundance, accounting for 91.32%, followed by the phylum Basidiomycota, with 8.64%. No significant difference was found between the PM_2.5_ and PM_10_ samples in winter or spring (*p* > 0.05). For each particle size class, differences in fungal phyla were analyzed across different seasons. The relative abundance of the phylum Ascomycota contained in PM_2.5_ in winter was greater than that in spring (*p* < 0.01) (Figure 6e). Conversely, the relative abundance of the phylum Basidiomycota in PM_2.5_ in spring was greater than that in winter (*p* < 0.01) (Figure 6f). Fungal genera with an average relative abundance greater than 0.01% are listed in Figure 7B. The different fungal genera present in PM_2.5_ and PM_10_ during the winter and spring seasons are shown in Supplementary Figure S4.

### 3.5. Potential Pathogens and Allergens in PM_2.5_ and PM_10_

**Figure 8:**
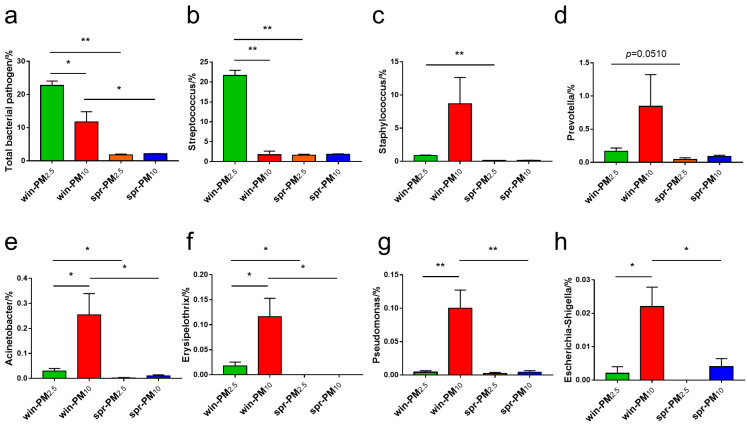
The relative abundance of potential bacterial pathogen genera are listed, including (**a**) total bacterial pathogen, (**b**) *Streptococcus*, (**c**) *Staphylococcus*, (**d**) *Prevotella*, (**e**) *Acinetobacter*, (**f**) *Erysipelothrix*, (**g**) *Pseudomonas*, and (**h**) *Escherichia-Shigella*. * *p* < 0.05, ** *p* < 0.01.

According to the directory of pathogenic microorganisms infecting humans from the Ministry of Health of the People’s Republic of China (MOHC), a total of seven pathogenic bacterial genera (*Streptococcus*, *Staphylococcus*, *Prevotella*, *Acinetobacter*, *Erysipelothrix*, *Pseudomonas*, and *Escherichia-Shigella*) were identified in all samples (Figure 8). In winter, the relative abundances of the total pathogenic bacteria and the genus *Streptococcus* in PM_2.5_ were significantly higher than those in PM_10_ (*p* < 0.05) (Figure 8a,b). The relative abundances of the genera *Acinetobacter*, *Erysipelothrix*, *Pseudomonas*, and *Escherichia-Shigella* in PM_10_ in winter were significantly higher than those in PM_2.5_ (*p* < 0.05) (Figure 8e–h). No significant differences in these potential pathogenic bacterial genera were found between PM_2.5_ and PM_10_ samples in spring (*p* > 0.05). In PM_2.5_, the relative abundances of total potential pathogenic bacteria genera, *Streptococcus*, *Staphylococcus*, *Acinetobacter*, and *Erysipelothrix* contained in winter were higher than those in spring (*p* < 0.05) (Figure 8a–c,e,f). In PM_10_, the relative abundances of total potential pathogenic bacteria genera, *Acinetobacter*, *Erysipelothrix*, *Pseudomonas*, and *Escherichia-Shigella* contained in winter were higher than those in spring (*p* < 0.05) (Figure 8a,e–h).

**Figure 9:**
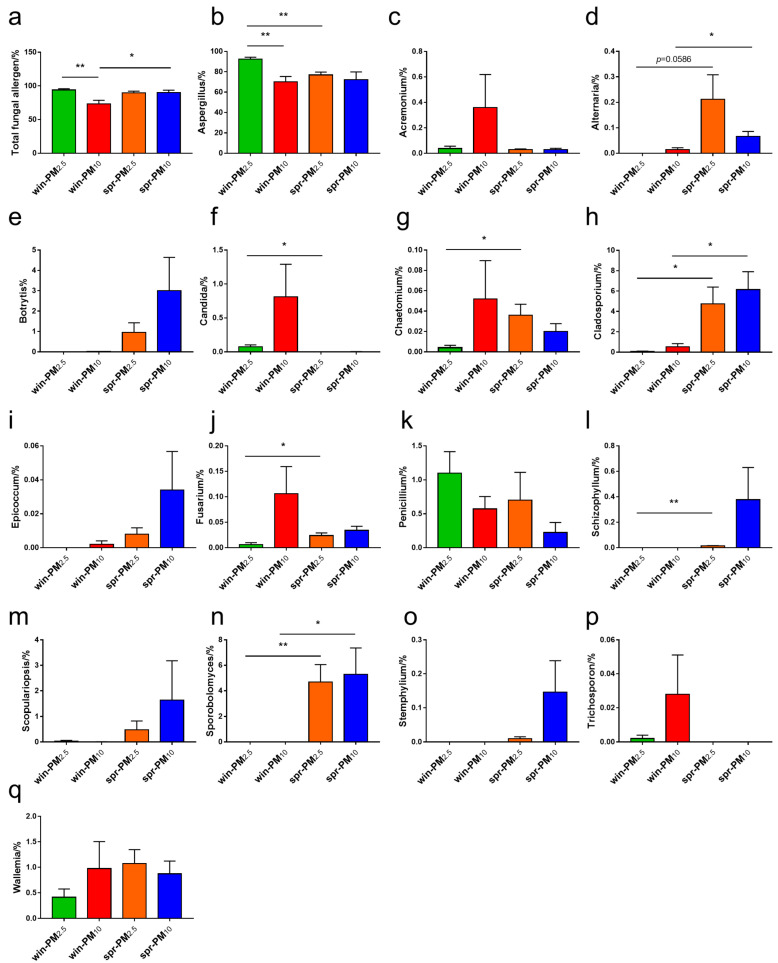
The relative abundance of potential fungal allergen genera, including (**a**) total fungal allergen, (**b**) *Aspergillus*, (**c**) *Acremonium*, (**d**) *Alternaria*, (**e**) *Botrytis*, (**f**) *Candida*, (**g**) *Chaetomium*, (**h**) *Cladosporium*, (**i**) *Epicoccum*, (**j**) *Fusarium*, (**k**) *Penicillium*, (**l**) *Schizophyllum*, (**m**) *Scopulariopsis*, (**n**) *Sporobolomyces*, (**o**) *Stemphylium*, (**p**) *Trichosporon*, and (**q**) *Wallemia*. * *p* < 0.05, ** *p* < 0.01.

From the 123 fungal allergen genera listed by Simon-Nobbe [19], a total of 16 fungal allergen genera (relative abundance >0.01%) were identified in the samples (Figure 9). As shown in Figure 9a,b, the relative abundances of the total fungal allergen genera and *Aspergillus* in PM_2.5_ in winter were higher than those in PM_10_ (*p* < 0.05). No significant difference in fungal allergen genera was found between the PM_2.5_ and PM_10_ samples in spring (*p* > 0.05). For each particle size class, differences in fungal phyla between seasons were analyzed. For PM_2.5_, the relative abundances of *Aspergillus* and *Candida* in winter were higher than those in spring (*p* < 0.05) (Figure 9b,f); however, the relative abundances of *Chaetomium*, *Cladosporium*, *Fusarium*, *Schizophyllum*, and *Sporobolomyces* in spring were higher than those in winter (*p* < 0.05) (Figure 9g,h,j,l,n). For PM_10_, the relative abundances of the total fungal allergen genera, *Alternaria*, *Cladosporium*, and *Sporobolomyces* in spring were higher than those in winter (*p* < 0.05) (Figure 9a,d,h,n).

## 4. Discussion

In this study, PM_2.5_ and PM_10_ from a pig barn were characterized via microscopic morphology and chemical content. The morphology of PM in this study was similar to that of previous findings [20], which indicates that the primary sources of PM in the pig barns are feed, feces, and skin or dander particles [21]. For airborne PM_2.5_ from the sow barns, 14.5% came from feed, 69.8% came from manure, 11.7% came from the skin, and 4.1% came from the outside. For the airborne PM_2.5–10_, 6.3% came from the feed, 84.1% came from the manure, 7.9% came from the skin, and 1.6% came from the outside [20]. For both PM_2.5_ and PM_2.5–10_, the main source of PM was feces, followed by feed and then skin. However, some studies have shown that the morphologies of fecal particles and feed particles are very similar because of the presence of undigested feed in the feces, which makes distinguishing between fecal particles and feed particles difficult. Therefore, we cannot entirely determine the sources of PM via scanning electron microscopy, but it can serve as a preliminary reference. In addition, 90% of PM from livestock barns is composed of organic matter [22]. In this study, the combined C and O contents in PM_2.5_ and PM_10_ were 67.6% and 77.21%, respectively. The PM_2.5_ and PM_10_ samples also contained Na, Mg, Al, Si, S, Cl, K, Ca, Ti, Fe, Zn, Ba, and W, which are commonly found in PM samples from livestock barns [23].

Next, we investigated the microbial composition of PM_2.5_ and PM_10_ from a pig barn during winter and spring. The results demonstrated that fungi and bacteria exhibited clear seasonal separation in their community structure between winter and spring. However, no significant differences were observed between PM_2.5_ and PM_10_ samples within the same season, suggesting that seasonality had a stronger influence on microbial assemblage in PM than particle size. This finding was consistent with our previous research on the microbial composition of PM_2.5_ from a nursery pig barn during all four seasons. In that study, the bacterial composition differed significantly among all seasons, except for autumn and summer, and the fungal composition differed among all seasons [15]. Jiang et al. [24] reported that the microbial compositions of PM samples from the atmosphere were significantly different in summer and winter, but no obvious differences were detected in relation to particle sizes (PM_1.0_, PM_2.5_, PM_10_) in the same season. This can be attributed to the environmental factors inside livestock barns. Temperature, relative humidity, and wind speed are important factors that influence airborne microorganisms. Generally, temperature and relative humidity are positively correlated with microbial growth [25]. However, different types of microorganisms have different requirements for temperature and humidity, which are context specific.

A total of 23 different bacterial phyla were identified in this study, among which Firmicutes had the highest relative abundance (96.40%), followed by Actinobacteria, Bacteroidetes, and Proteobacteria. These results are consistent with those of previous studies. Dai et al. reported that Firmicutes, Actinobacteria, Bacteroidetes, and Proteobacteria were the main bacterial phyla in PM_2.5_ from poultry barns. Firmicutes and Actinobacteria accounted for more than 80% of the total bacteria [26]. Wang et al. reported that airborne PM_2.5_ samples collected inside a fattening pig barn contained primarily Firmicutes, followed by Bacteroidetes, and Proteobacteria [27]. In contrast, in residential areas, Proteobacteria is the most abundant bacterial phylum [28].

Seven different fungal phyla were identified. The total relative abundance of Ascomycota and Basidiomycota was 99.96%, and the relative abundance of Ascomycota was the highest (91.32%). Tang et al. reported that the total relative abundance of Ascomycota and Basidiomycota in PM_2.5_ from a nursery pig barn was 79.5% over one year [15]. Dai et al. reported that the total relative abundance of Ascomycota and Basidiomycota in PM_2.5_ from a poultry barn exceeded 80% [26]. Additionally, Jiang et al. reported that the average relative abundance of Ascomycota contained in particles of different sizes was 62.42% [24]. The above results indicate that Ascomycota and Basidiomycota are the major fungal phyla present in airborne PM.

The presence of potentially pathogenic bacterial genera and allergenic fungal genera in the air of pig barns affects the health of both the animals and the workers. We identified these potentially pathogenic bacterial genera and allergenic fungal genera in the PM_2.5_ and PM_10_ samples during different seasons. According to the directory of pathogenic microorganisms infecting humans from the MOHC, the following seven potential pathogenic bacterial genera were identified in all samples: *Streptococcus*, *Staphylococcus*, *Prevotella*, *Acinetobacter*, *Erysipelothrix*, *Pseudomonas*, and *Escherichia-Shigella*. Pathogenic bacteria affect the health of animals and farm workers and present major challenges to the biosecurity and control of livestock farms [29,30]. *Streptococcus* is an important zoonotic pathogen. It can cause septicemia, meningitis, and arthritis through mucosal infection and even acute death in severe cases. It can also induce various types of respiratory tract inflammation [31]. *Escherichia-Shigella* is a common pathogen that causes diarrhea in piglets [32]. *Staphylococcus*, as a conditional pathogen, is widely found in the air of livestock barns and on the surfaces of animals, and it can induce diseases, such as exudative dermatitis, respiratory tract infection, meningitis, and septicemia [33,34]. To induce disease, potentially pathogenic species must possess essential virulence-associated genes. The pathogenic nature of bacteria, dictated by their virulence, varies from non-virulent to highly virulent strains, depending on particular DNA segments that encode virulence factors [35,36]. These factors encompass proteins involved in adhesion, invasion, toxin production, immune evasion, and biofilm development [37]. The outcome of bacteria–host interactions is influenced by both the host’s defense mechanisms and the bacteria’s virulence capabilities. Further research can focus on the toxicity of specific species within the potentially pathogenic bacterial genera identified in PM.

From the list of 123 fungal allergen genera reported by Simon-Nobbe [19], a total of 16 fungal allergen genera (relative abundance >0.01%) were identified in all samples, including *Aspergillus*, *Acremonium*, *Alternaria*, *Botrytis*, *Candida*, *Chaetomium*, *Cladosporium*, *Epicoccum*, *Fusarium*, *Penicillium*, *Schizophyllum*, *Scopulariopsis*, *Sporobolomyces*, *Stemphylium*, *Trichosporon*, and *Wallemia*. Among them, *Aspergillus*, *Fusarium*, and *Scopulariopsis* are the most common fungal genera in pig barns [38]. In this study, the fungal genus with the highest relative abundance in PM_2.5_ and PM_10_ was *Aspergillus*, accounting for more than 70% of fungal genera. If a high concentration of *Aspergillus* is repeatedly inhaled, it can cause respiratory diseases such as allergic asthma, rhinitis, and pneumonia [19]. Aflatoxin, a secondary metabolite produced by related strains of *Aspergillus*, causes mold and rot in corn, peanuts, and cottonseed, is a carcinogen, and can cause liver damage in humans and animals [39]. Fusarine, which is produced by *Fusarium*, is highly toxic and can inhibit the growth and development of animals, damage the immune system and intestinal mucosal barrier, and cause DNA damage [32]. *Scopulariopsis* is a saprophytic pathogen that can cause dermatomycosis, keratitis, and lung damage [40].

Finally, it is worth noting that, according to the definitions of PM_10_ and PM_2.5_, PM_2.5_ is a subset of PM_10_. Therefore, in principle, PM_10_ samples should contain all PM_2.5_ components, and it is possible to detect certain biological signatures in PM_10_ that are absent in PM_2.5_. However, if any biological signatures appear in PM_2.5_ but are absent in PM_10_ during our analysis, this discrepancy must be attributed to uncertainties in the measurement method.

## 5. Conclusions

The β diversity results revealed that season, rather than the particle size, significantly influenced the microbial community composition in the PM samples. In total, seven pathogenic bacterial genera and 16 fungal allergen genera were identified in particle samples across different seasons. In winter, the relative abundances of total bacterial pathogens and fungal allergens in PM_2.5_ were both higher than those in PM_10_, suggesting that PM_2.5_ in winter may pose a greater threat to animals and humans. In addition, for both PM_10_ and PM_2.5_, the relative abundance of total bacterial pathogens in winter was significantly higher than that in spring, which suggests that PM samples from winter pose a greater risk than those from spring. Our research provides a foundation and serves as a valuable reference for analyzing the characteristics of different-sized PM samples across different seasons. However, our study has several limitations. Future research should focus on airborne PM from diverse types of livestock barns, rather than a single pig barn, in order to obtain more representative data.

## Figures and Tables

**Table 1:** Chemical composition of PM_10_ and PM_2.5_ in different seasons (mass percentage %).

	PM_10_ in Winter			PM_2.5_ in Winter			PM_10_ in Spring			PM_2.5_ in Spring		
	Max	Min	Mean	Max	Min	Mean	Max	Min	Mean	Max	Min	Mean
O	44.7	40.73	42.35	48.75	39.9	43.68	40.47	36.78	38.78	35.6	28.9	32.41
C	37.98	32.02	34.86	29.65	21.17	23.92	37.1	19.05	29.59	41.24	27.37	35.89
Si	8.72	6.89	7.73	18.48	11.64	15.52	19.26	8.04	12.26	19.82	8.61	12.52
N	5.16	2.08	4.01	-	-	-	3.51	1.81	2.67	4.54	2.99	3.76
Al	2.51	2.1	2.28	2.28	1.88	2.09	2.26	1.1	1.55	2.4	1.3	1.62
K	3.5	0.88	1.62	1.47	1.06	1.26	1.73	1.04	1.42	2.27	1.08	1.61
Mg	1.76	0.98	1.45	1.19	0.18	0.48	0.8	0.2	0.4	0.5	0.15	0.3
Ca	1.57	1.11	1.37	2.71	1.12	1.8	2.55	1.54	2.1	2.72	1.23	1.86
Na	1.64	0.74	1.1	5.02	3.13	4.31	4.38	2.24	3.13	3.53	2.33	2.91
Zn	0.81	0.64	0.71	3.02	1.78	2.31	4.37	1.84	2.59	4.98	1.88	3.02
P	0.89	0.41	0.68	-	-	-	0.87	0.31	0.53	0.73	0.27	0.46
W	0.62	0.55	0.58	1.45	1.4	1.42	1.67	0.86	1.26	0.81	0.79	0.80
Ba	0.74	0.19	0.46	4.07	2.42	3.09	5.51	2.16	3.3	6.42	2.37	3.73
Fe	0.46	0.35	0.41	0.25	0.08	0.16	0.39	0.2	0.3	0.45	0.22	0.31
S	0.35	0.3	0.33	0.8	0.32	0.47	1.69	0.71	1.13	1.24	0.65	0.81
Cl	0.37	0.25	0.32	0.25	0.13	0.17	0.67	0.18	0.36	0.49	0.28	0.4
Ti	-	-	0.08	-	-	0.12	-	-	0.18	-	-	-

Note: - means no detection.

## Data Availability

The datasets generated and/or analyzed during the current study are available from the corresponding author upon reasonable request.

## Supplementary Materials

The following supporting information can be downloaded at: https://www.mdpi.com/article/10.3390/ani15081116/s1, Figure S1: The floor plan of the pig house with sampling point marked; Figure S2: Microscopic morphology and energy dispersive spectroscopy (EDS)-chemical composition of PM_2.5_ in winter, PM_10_ in winter, PM_2.5_ in spring and PM_10_ in spring; Figure S3: The differential bacterial genera contained in PM_2.5_ and PM_10_ during the winter and spring seasons; Figure S4: The differential fungal genera contained in PM_2.5_ and PM_10_ during the winter and spring seasons.

## Abbreviations

The following abbreviations are used in this manuscript:

MOHC	Ministry of Health of the People’s Republic of China
NMDS	nonmetric multidimensional scaling
PCoA	principal coordinate
PM	particulate matter
PM2.5	fine particulate matter
