# Supplementary material for: Seasonal Dynamics of Microbial Communities in PM2.5 and PM10 from a Pig Barn
Source: Animals (Basel). 2025 Apr 12;15(8):1116. doi: 10.3390/ani15081116 (PMC12024127; doi:10.3390/ani15081116)
Supplement: Supplementary file 1 [file animals-15-01116-s001.zip › animals-3519639-supplementary.pdf]

Supplementary Figure S1

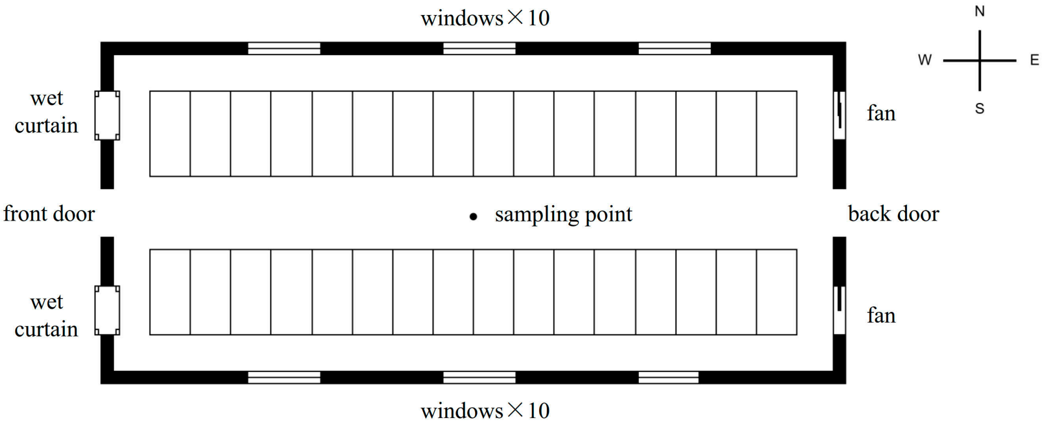

Supplementary Figure S1 The floor plan of the pig house with sampling point marked.

Supplementary Figure S2

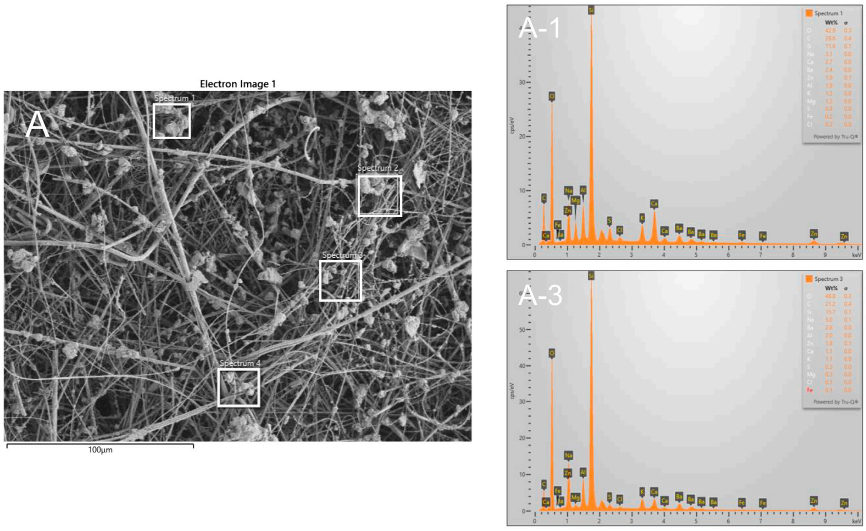

Microscopic morphology (A) and energy dispersive spectroscopy (EDS)-chemical composition (A1-A4) of $PM_{2.5}$ in the pig house during the winter season.

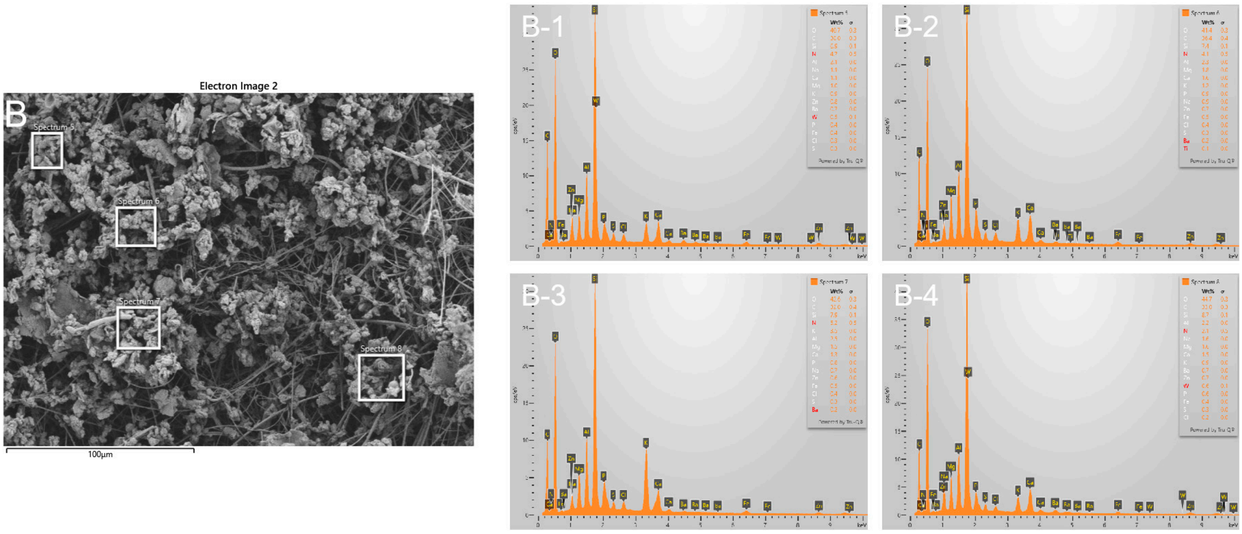

Microscopic morphology (B) and energy dispersive spectroscopy (EDS)-chemical composition (B1-B4) of PM_{10} in the pig house during the winter season.

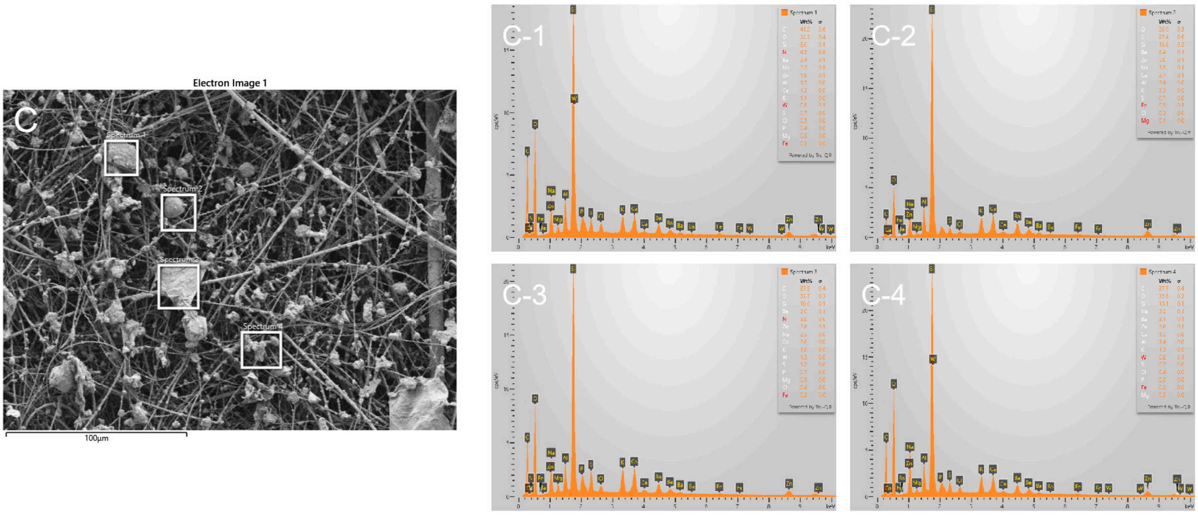

Microscopic morphology (C) and energy dispersive spectroscopy (EDS)-chemical composition (C1-C4) of $PM_{2.5}$ in the pig house during the spring season.

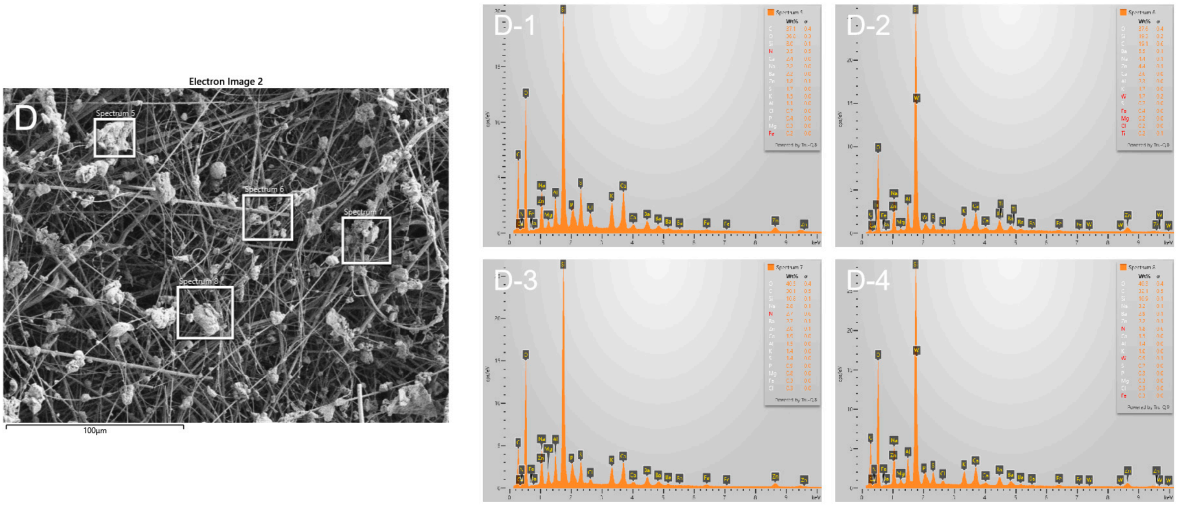

Microscopic morphology (D) and energy dispersive spectroscopy (EDS)-chemical composition (D1-D4) of $PM_{2.5}$ in the pig house during the spring season.

Supplementary Figure S3

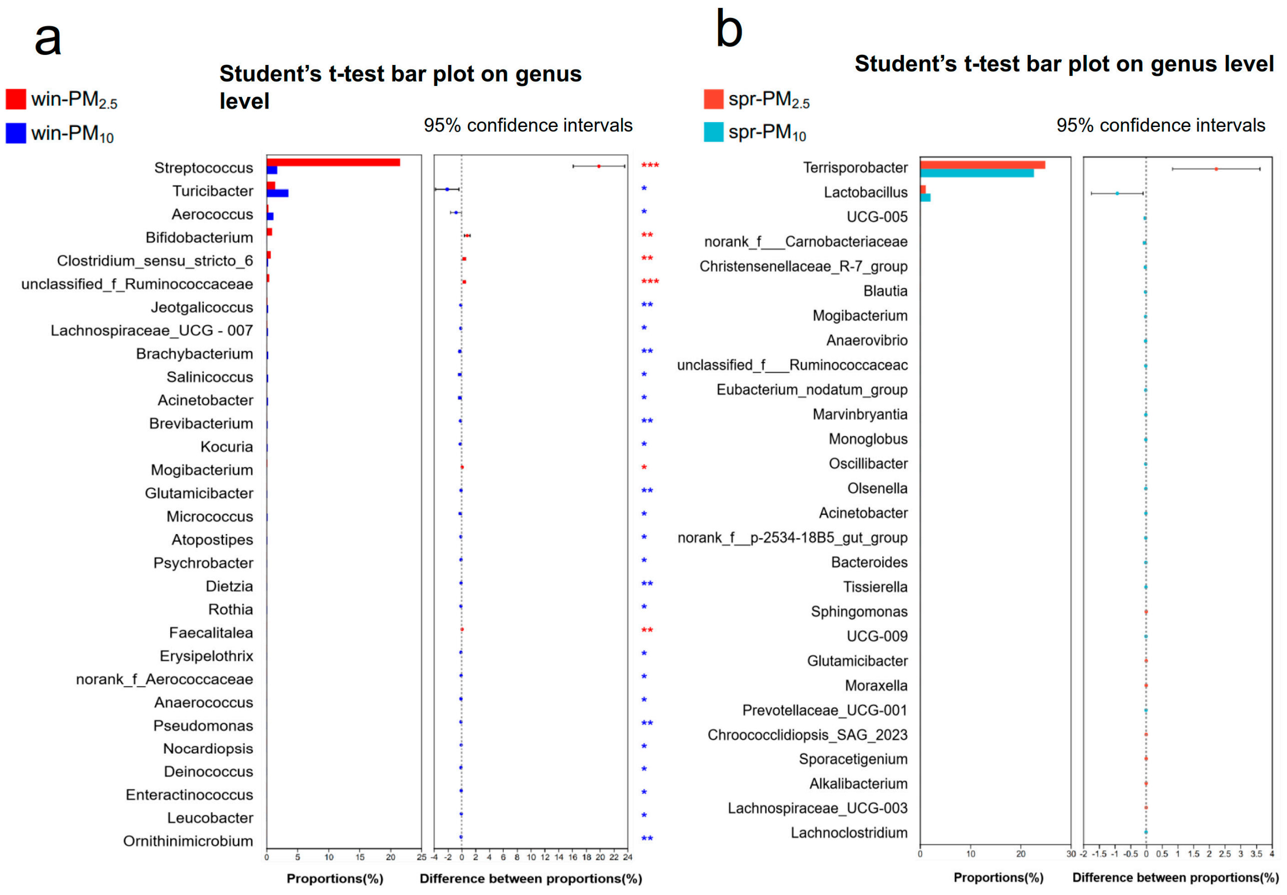

Supplementary Figure S3 The differential bacterial genera contained in $PM_{2.5}$ and PM_{10} during the winter (a) and spring (b) seasons. The top 30 bacterial genera ranked by relative abundance were listed. *p<0.05，**p<0.01.

Supplementary Figure S4

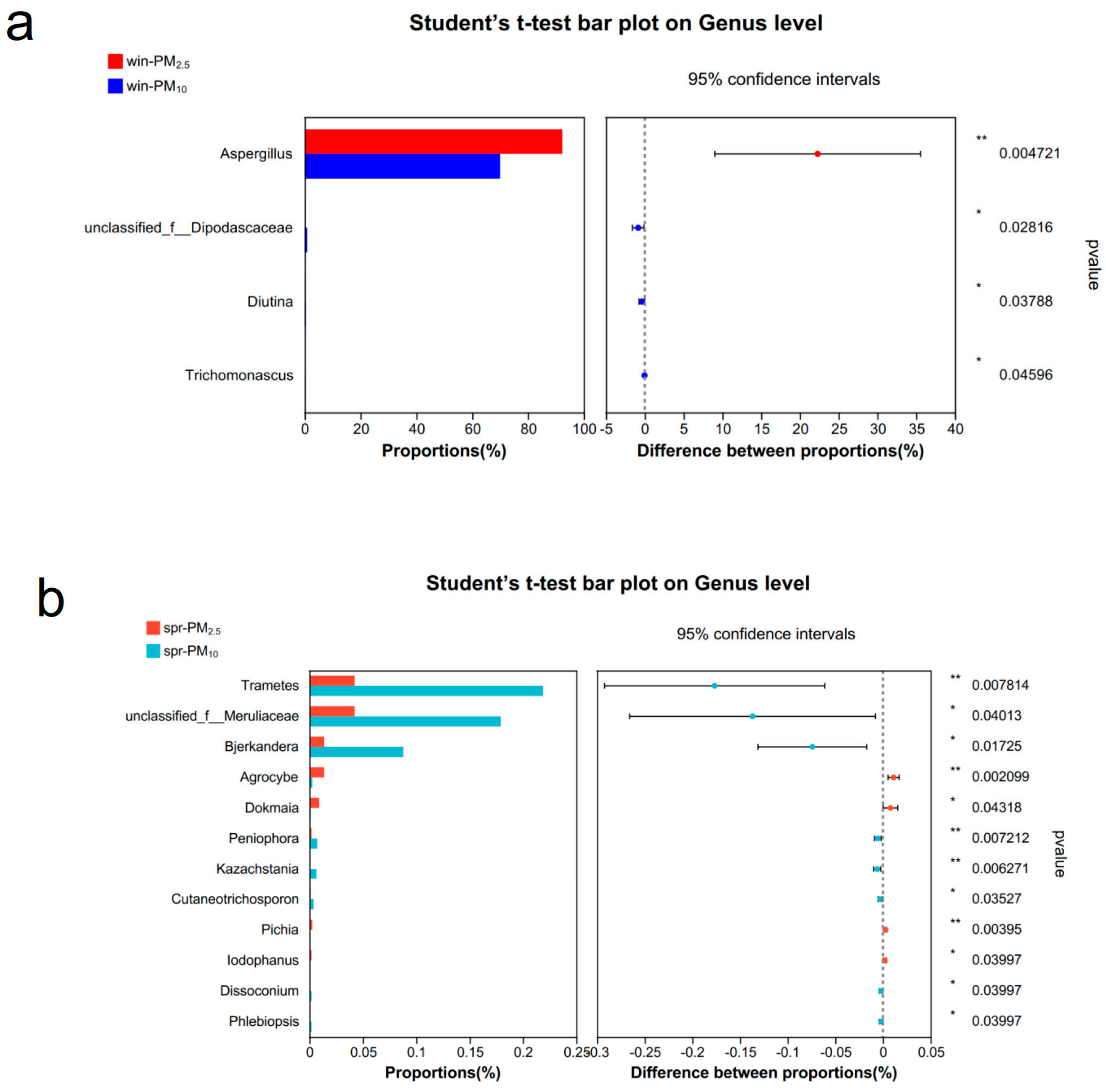

Supplementary Figure S4 The differential fungal genera contained in $PM_{2.5}$ and PM_{10} during the winter (a) and spring (b) seasons. The top 30 fungal genera ranked by relative abundance were listed. *p<0.05，**p<0.01.
